# Middle-aged death and taxes in the USA: Association of state tax burden and expenditures in 2005 with survival from 2006 to 2015

**DOI:** 10.1371/journal.pone.0214463

**Published:** 2019-04-12

**Authors:** Todd A. MacKenzie, Jason Houle, Steven Jiang, Tracy Onega

**Affiliations:** 1 Department of Biomedical Data Science, Dartmouth College, Lebanon, NH, United States of America; 2 Department of Sociology, Dartmouth College, Hanover, NH, United States of America; Emory University School of Public Health, UNITED STATES

## Abstract

**Background:**

Longevity in the United States ranks below most other Western nations despite spending more on healthcare per capita than any other country. Across the world, mortality has been declining, but in the USA the trend toward improvement has stalled in some middle-aged demographic groups. Cross-national studies suggest that social welfare is positively associated with longevity. The United States has less government sponsored welfare, education and healthcare than almost all other Western nations, but the level of this social welfare commitment varies across the states. In this study we examined the association of state tax burden and state government expenditures with subsequent middle-aged mortality.

**Methods:**

The primary exposure was state tax burden in 2005, defined as proportion of all state income paid to the state. We also examined the impact of state expenditures per capita in 2005 for education, healthcare, welfare, police and highways. The dependent variable was mortality during the subsequent 10 years. Death counts and population sizes by sex, age group and race strata for 2006–2015 were abstracted from CDC WONDER. Binomial logistic regression was employed based on the number of deaths and underlying population within each county-sex-age-race bin.

**Results:**

State tax burden in 2005 varied from 5.8% to 12.2%. An increase of 1.0 percentage point in state tax burden was associated with a 5.8% (SE = 0.1%) reduction in mortality adjusted for sex, age and race, but was associated with a 1.1% (SE = 0.1%) reduction when further adjusting for state income and education levels. Controlling for sex, age and race each type of state expenditures was associated with decreases in middle aged mortality, notably K-12 education (reduction of 4.7%, SE = 0.1%, per 10% expenditure increase) except healthcare but all types were associated with mortality decreases further controlling for state income and education.

**Conclusion:**

The residents of states with higher state taxation and higher expenditures per capita have lower middle aged mortality rates.

## Introduction

Longevity in the United States (USA) ranks below most other Western nations despite spending more on healthcare per capita than any other country. During the past century, mortality has reduced around the world, but, in the USA, mortality trends have stalled and even reversed in lower education middle-aged whites [1], lower education females [2], as well as young adult white females [3]. The mortality rate varies across the 50 states, [4] [5] which may be related to state policies [6].

Mortality is associated with income in the USA [7] [8] [9]. The income-mortality association may be due to access to healthy food, safer, less stressful and less polluted environments, and access to healthcare. Governments fund these goals using taxation. The income-mortality gradient which is characterized by diminishing returns (e.g., impact of $10,000 on a low income earner exceeds the impact of $10,000 on a high income earner) implies that redistribution of income from the rich to the poor increases longevity in the aggregate [10]. In the USA, federally funded programs include universal Medicare and Social Security, for the elderly, and means-tested healthcare and income assistance (i.e. redistribution), for those younger than 65. Longevity increases with education level [11]. The disparity in mortality between the most and least educated has been growing for decades[12].

State government expenditures on education, social welfare, cash assistance, safety and healthcare vary across the United States and depend on state levied taxation. Proponents of tax reductions argue that lower taxes increase the ability of each individual to improve their own outcomes, such as longevity, and therefore improve outcomes overall, e.g. life expectancy of a state. The variation of tax burdens and government expenditures across states provides an opportunity to explore their association with mortality. State government expenditures on welfare and education have been linked to reduced mortality using a cohort study [13]. Spending on welfare relative to health expenditures has been linked to a number of health outcomes [14]. We hypothesized that higher state tax burdens and higher expenditures would be associated with lower mortality among middle-age individuals. We restrict our study to individuals under the age of 65 because federally funded Medicare and Social Security are universally available to the elderly. To address this hypothesis we examined the association of middle aged mortality in the 10 year period 2006–2015 with the state tax burdens and expenditures in the year 2005. We consider income tax, sales tax, corporate tax, an overall measurement of state tax burden, and expenditures on education, welfare, police, highways and health.

## Methods

Population: All residents in the age range 40 to 64 residing in one of the 50 States or the District of Columbia during the years 2006–2015.

Outcome: Death from all causes.

Exposure: The primary exposure of interest is the *state and local tax burden* in 2005, as calculated by the proportion of overall state income that goes to state or local taxes [15]. Secondary exposures included 2005 metrics of state income tax burden, state sales tax rate, and corporate tax, as well as slope of tax burden and income tax across quintiles of income, and state and local government expenditures per capita in each state on education, welfare, health and hospitals, highways and police (REF).

Covariates: Race (white, black, Asian or Pacific Islander, other), gender, age (in years grouped as 40–44, 45–49, 50–54, 55–59 and 60–64), state level proportion of residents over age 24 with a high school education and with a bachelor’s degree, state median income, and state income per capita.

Data Sources: CDC WONDER [16] was used to extract a table of number of deaths and census counts by state, gender, age group and race. Tax burden by state in 2005 was taken from the Tax Foundation [15], income and corporate tax and tax burden by quintile from the Institute on Taxation and Economic Policy “Who Pays?” report in 2003 [17], sales tax rate in 2005 from the Tax Policy Center [18]. Expenditures by state government in 2005 were obtained from the Tax Policy Center[19].

## Statistical analysis

Mortality data from CDC WONDER was extracted as number of deaths (numerator), and census population-years (denominator) stratified by state, sex, race (White, Black, Asian, other) and age (in 5 years age groups, the smallest granularity using CDC WONDER). We modelled the annual mortality rate using binomial logistic regression (e.g. a binomial distribution is assumed for the number of deaths in a given state-sex-race-age strata given the population size of that strata). All models controlled for gender, race and age (5 year grouping) as a three dimensional factor (i.e. controlling for the 2x4x5 = 120 level factor resulting from 2 levels of gender, 5 age groups and 4 levels of race), in order to avoid parametric modelling assumptions with respect to these demographics.

We estimated the odds ratio relating annual mortality to each tax and state expenditure, one at a time, using two models (i.e. two approaches to adjustment). The odds ratio can be regarded a ratio of risks, i.e. mortality ratio, because mortality is a low frequency event. The first model includes only sex, race, and age as described above. The second model controlled for state median income, per capita income, proportion with high school diplomas, proportion with bachelor degrees, and each of their interactions with the 120 level sex-age-race factor, in order to allow for the possibility that any effects of state education and state income are modified by age, sex and/or race. The impact of taxation levels is measured as the mortality ratio per percentage point increase in tax (e.g. 5% to 6%) while the impact of expenditure levels is measured in 10% increases in expenditures per capita (e.g. $500 to $550 or $2000 to $2200) which is accomplished by transforming expenditures by the logarithm with base of 1.10. In secondary analyses, we examined the effect of tax burden in subgroups stratified by gender, race and presence of income tax.

The scatterplots of adjusted mortality versus the state taxes and expenditures were calculated using observed mortality in a state divided by expected mortality, where expected mortality was calculated using sex, age and race, according to a binomial logistic regression model as specified above.

## Results

Table 1 presents descriptive statistics for taxation levels and state and local government expenditures in 2005 (or 2003), as well income and education level across the 50 states and DC. Tax Burden varied from 5.8% in Alaska to 12.2% in New York. The tax burden of individuals in the lowest quintile of income varied from 3.8% to 17.6%, while the tax burden in the highest income quintile varied from 2.5% to 10.6%. Income tax rates varied from 0, for all individuals in 7 states, to as high as 8.4%, for the highest tax bracket. In 2005, sales tax varied from 0% (in 4 states) to 7%, with a median of 5%. Due to the exemption of food from sales tax in some states, the median sales tax on food was 0% with a high of 7% in two states. Expenditures on K-12 education per state varied from $1,168 per capita to $2,593, while per capita expenditures on post-secondary education varied from $180 to $981. Welfare expenditures varied 4 fold from $673 to $3,106.

**Table 1:** Distribution of state tax and other characteristics in 2005.

	Minimum	Median	Maximum
Median Income in 2005	$32,875	$44,993	$63,368
Per Capita Income in 2003	$23,448	$29,944	$48,342
High School Education %	7.8	8.7	9.3
Bachelors Degree Education %	1.5	2.6	4.7
**Taxation Levels**			
Tax Burden %	5.8	9.6	12.2
Income Tax Burden %	0.0	3.6	5.8
Sales Tax %	0.0	5.0	7.0
Food Sales Tax %	0.0	0.0	7.0
Corporate Tax Burden %	0.0	6.5	10.0
**Expenditures Per Capita**			
Education K-12	$1,168	$1,496	$2,593
Education Post Secondary	$180	$630	$981
Welfare	$673	$1,106	$3,106
Health and Hospitals	$139	$480	$1,610
Highways	$140	$420	$1,802
Police	$123	$215	$769

From 2006 to 2016, annual middle-aged mortality ranged from 6 per 10,000, in Asians or Pacific Islanders females aged 40–44, to 202 per 10,000, in Black males aged 60–64. Males were at 66% more risk of death than females. Compared to middle-aged Whites, Asian or Pacific Islanders had 54% less risk, Blacks had 58% more risk, American Indian of Alaska Native were at 4% more risk. Compared to those aged 40–44, the multiplicative increase in risk was 1.55 times, 2.38, 3.47 and 4.04 among 45–49, 50–54, 55–59 and 60–64 year olds, respectively. Middle-aged mortality dropped 1.8% (0.1%) for every $1000 increase in state median income, and 2.8% (0.1%) for every $1000 increase in average income. An increase in the proportion of high school and college educated residents was associated with 1.5% (0.1%) and 2.5% (0.1%) reductions in mortality, respectively.

**Fig 1:**
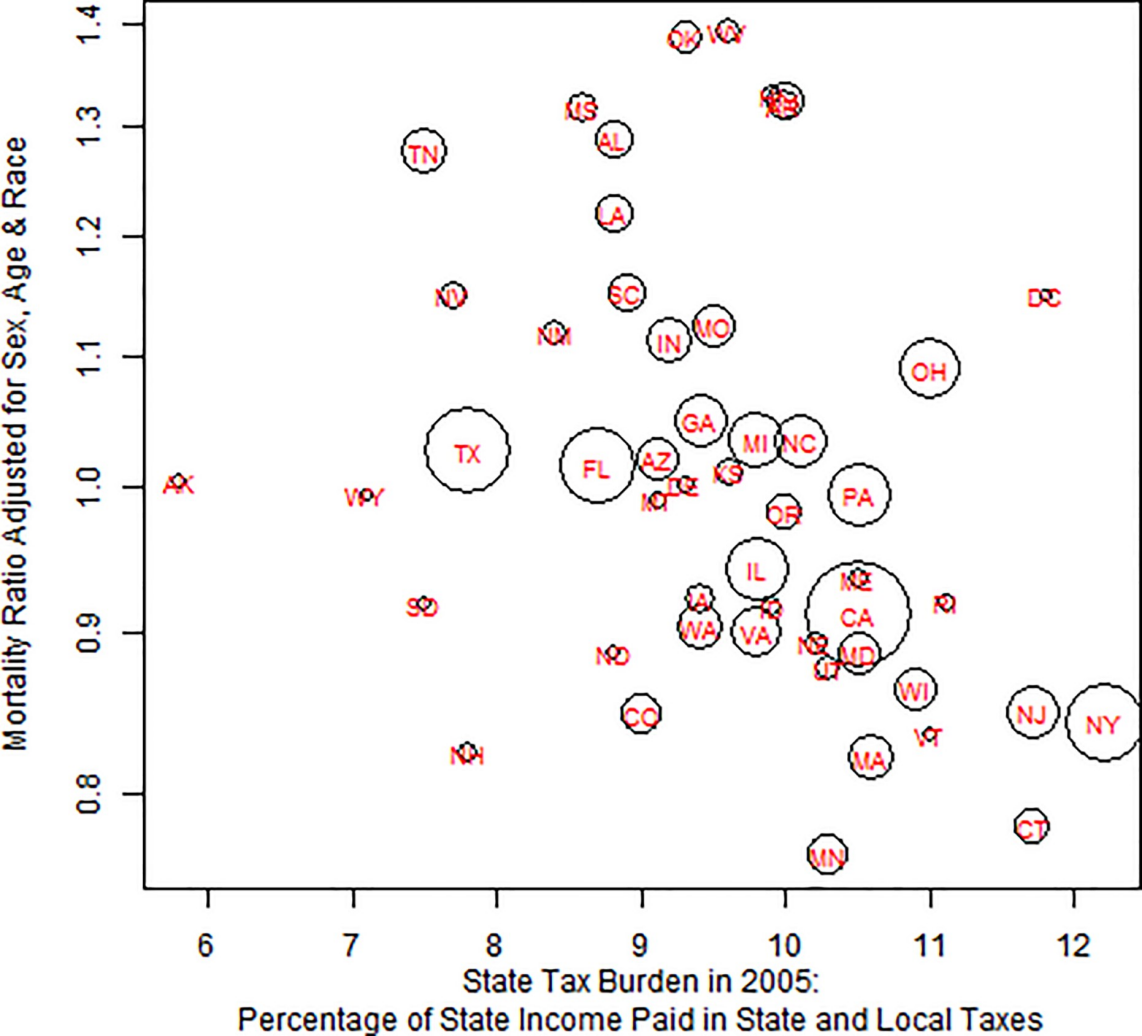
The association of state tax burden (percentage of state income paid in state and local taxes) in 2005 with middle-age mortality adjusted for race, gender and 5 year age groups. Area of circle is proportional to population size.

Fig 1 represents variation between the 50 states and DC in middle-age mortality, adjusted for the distribution of age, race as well as gender in each state. Compared to average middle-age mortality in the USA, rates were 25% or higher in several United States. Hawaii was also at increased risk, which may be due to the grouping of Asians and Pacific Islanders. Residents of northern states had 15% less middle-aged mortality.

**Fig 2:**
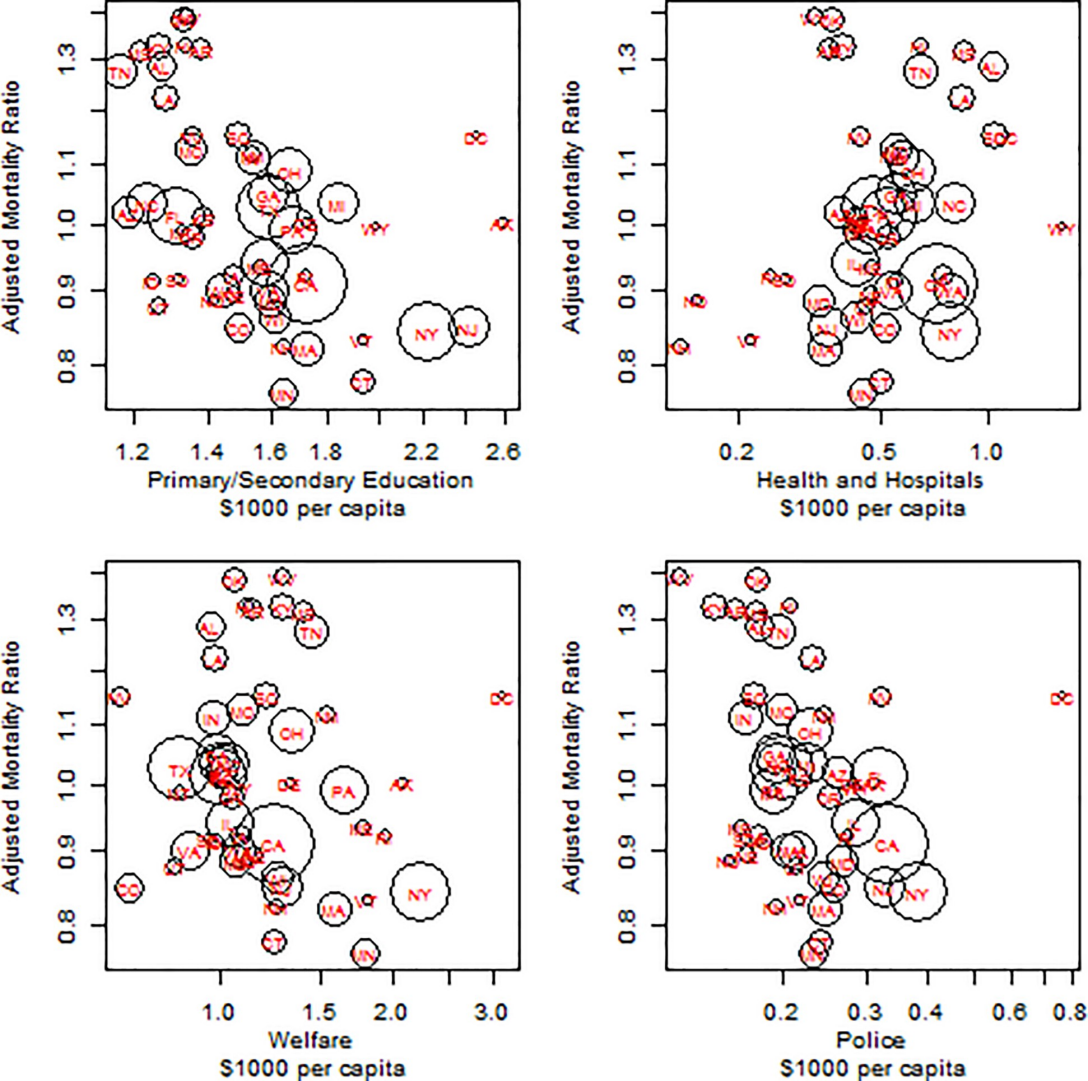
The association of state expenditures in 2005 for primary and secondary education (pane a), health and hospitals (pane b), welfare (pane c) and police (pane d) with middle-age mortality adjusted for race, gender and 5 year age groups. Area of circle is proportional to population size.

The top rows of Table 2 presents the change in middle-age mortality associated with an increase of one percentage point in various tax burdens. Each increase of one percentage point in tax burden was associated with a 5.8% (0.1%) reduction in mortality controlling for race, age and gender, as also shown in Fig 2. The corresponding reduction in mortality was 1.1% when we also controlled for state income and education. The state income tax rate had a considerably smaller association with mortality. There was no association of corporate tax burden with mortality controlling for state income and education. The state sales tax was associated with a small increase in mortality. However, each percentage point increase in the sales tax paid on food was associated with a 2.7% (0.1%) increase in middle-age mortality and a 1.2% (0.1%) increase when controlling for state income and education, as well as race, sex and age.

**Table 2:** Association with middle-age mortality of state taxation and government expenditures.

	Association adjusted for sex, age and race			Adjusted also for state income and education		
Tax Burden in 2005 (% Point)	% Change	95% C.I.		% Change	95% C.I.	
Overall Tax Burden	-5.8	-5.9	-5.7	-1.1	-1.1	-1.0
Income Tax Burden	-1.5	-1.5	-1.4	0.1	0.0	0.2
Corporate Tax Burden	-1.4	-1.4	-1.4	0.0	0.0	0.1
Sales Tax	1.0	0.9	1.0	0.1	0.0	0.2
Sales Tax on Food	2.7	2.7	2.8	1.2	1.2	1.3
**State Expenditures (10% Increase)**						
Education K-12	-4.7	-4.7	-4.6	-1.5	-1.5	-1.4
Education Post Secondary	0.0	0.0	0.1	-0.7	-0.7	-0.6
Welfare	-1.3	-1.3	-1.2	-0.2	-0.2	-0.1
Health and Hospitals	0.6	0.6	0.6	-0.2	-0.2	-0.1
Highways	-0.9	-1.0	-0.9	-1.6	-1.6	-1.5
Police	-2.9	-3.0	-2.9	-0.7	-0.7	-0.6

The association of state tax burden with mortality was not modified by gender (P>0.10). The association did vary across races (P<0.0001). Controlling for state income and education, as well as gender and age, each 1% increase in state tax burden, was associated with a mortality decrease of 1.0% (0.1%) in Whites, 0.7% (0.2%) in Blacks, 10.1% (0.7%) in First Nations, but a 3.6% (0.7%) increase in mortality in Asians or Pacific Islanders.

The lower rows of Table 2 report the association of state government expenditures per capita with middle age mortality. A 10% increase in primary and secondary education is associated with a 4.7% reduction (95%CI: 4.6–4.7) in mortality adjusted for sex, race and age, and a 1.5% reduction if the model incorporates state income and education levels. Expenditures on post-secondary education were not associated with middle age mortality unless one controlled for state income and education. A 10% increase in welfare expenditures was associated with a 0.2% reduction in middle age mortality controlling for sex, age, race, state income and state education. There was no clear association of health and hospitals expenditures with middle age mortality. Increased expenditures on highways and police were associated with decreased mortality.

## Discussion

We have shown an association of state tax burden and subsequent middle age mortality using the 10 year period 2006–2015. While we found no association of state sales tax with mortality, we did find that a sales tax on food is related to increased mortality. Increasing state expenditures were associated with reductions in middle age mortality, especially expenditures for K-12 education. Our study is the first to our knowledge that links mortality to state tax and expenditures using data on all middle aged deaths in the USA.

State taxation can be applied to sales, individual and corporate income as well as property and other forms, but our study does not address the joint effects of each form of taxation. We focused on an aggregate of these various forms known as tax burden [8] [17]. We did look at income tax rate and sales tax separately, but our findings were not as strong, with the exception of sales tax on food.

Sales taxes are a regressive form of taxation because less affluent individuals spend a higher proportion of their income compared to wealthier individuals. Conversely, the income tax brackets in many states are progressive. We examined the association with mortality of progressivity of the taxation, as measured by the slope of tax burden across quintiles, but the findings were null.

Our finding, that increased state taxation is associated with decreased state mortality, should not be interpreted at the individual level. Findings from a study of the income-longevity gradient in the USA [9] suggest that longevity increases as income increases, with a steep gradient at lower incomes and small gradient at high incomes. This suggests that the effect of taxation at the individual level is a longevity reduction. However, tax revenue provides funding that benefits the common good, such as safer and healthier environments and access to healthcare, which reduce mortality overall.

We found that as state tax burden increases, mortality in the state is reduced, in Whites, Blacks and Native Americans, but in Asians and Pacific Islanders mortality increases with state tax burden. One possible contributor to this reverse finding may be due to higher incomes among Asian Americans [20] and that individual income has a causal impact on individual longevity; hence, in the aggregate they may experience a loss of income and years of life that at is not offset by longevity gained due to government programs and infrastructure.

We control for state education level and state income as they are known to be positively associated with longevity and because more affluent states tend to higher taxation and higher expenditures. If higher education levels are a causal outcome of higher taxation and expenditures on education, then the associations we report that control for education level bias are estimates of association toward the null.

There are several ways in which the mortality versus tax associations we report may be confounded. It is in the personal interest of individuals living in states with high state taxes to move to states with low taxes. Since healthier and affluent individuals may find it easier to relocate, our estimates may be biased toward showing an association of lower taxation and reduced mortality. Tax revenue is also used to pay for state debt, which may be exacerbated in less affluent states and would bias estimates toward an association of state taxation and increased mortality.

Our findings that expenditures are associated with reduced mortality may be confounded. States with higher expenditures on healthcare, some of which are federal transfers for Medicaid, may spend because their population is less healthy and therefore shorter living. States with higher spending on social welfare, some of which is federal transfers for Temporary Assistance for Needy Families, may spend because they have a higher proportion of poor residents which is associated with higher mortality. Both of these pathways would bias toward findings that taxation and expenditures increase mortality.

In this era of American mediocrity in longevity and the recent Federal tax cut, the role of state government expenditures on population mortality and morbidity requires scrutiny. If the federal government reduces expenditures on health, welfare and education, mortality may increase (9]. State governments may want to increase financial commitment to health, welfare and education. More work is needed to determine the relative contributions of each type of expenditure on mortality and other health outcomes. The optimal distribution of expenditures may depend on the state and temporal factors, in which case the driving factor that influences a state government’s beneficial effect on its residents may be overall revenue.
